# Hydrogels to Support Transplantation of Human Embryonic Stem Cell-Derived Retinal Pigment Epithelial Cells

**DOI:** 10.3390/brainsci12121620

**Published:** 2022-11-25

**Authors:** Ying Wei, Uwimana Alexandre, Xiang Ma

**Affiliations:** Department of Ophthalmology, The First Affiliated Hospital of Dalian Medical University, Dalian 116014, China

**Keywords:** hESC-RPE, transplantation, hydrogel, retinal degenerative diseases, ARPE-19

## Abstract

Purpose: Retinal pigment epithelial (RPE) cells are highly specialized neural cells with several functions essential for vision. Progressive deterioration of RPE cells in elderly individuals can result in visual impairment and, ultimately, blinding disease. While human embryonic stem cell-derived RPE cell (hESC-RPE) growth conditions are generally harsher than those of cell lines, the subretinal transplantation of hESC-RPE is being clinically explored as a strategy to recover the damaged retina and improve vision. The cell-adhesion ability of the support is required for RPE transplantation, where pre-polarized cells can maintain specific functions on the scaffold. This work examined four typical biodegradable hydrogels as supports for hESC-RPE growth. Methods: Four biodegradable hydrogels were examined: gelatin methacryloyl (GelMA), hyaluronic acid methacryloyl (HAMA), alginate, and fibrin hydrogels. ARPE-19 and hESC-RPE cells were seeded onto the hydrogels separately, and the ability of these supports to facilitate adherence, proliferation, and homogeneous distribution of differentiated hESC-RPE cells was investigated. Furthermore, the hydrogel’s subretinal bio-compatibility was assessed in vivo. Results: We showed that ARPE-19 and hESC-RPE cells adhered and proliferated only on the fibrin support. The monolayer formed when cells reached confluency, demonstrating the polygonal semblance, and revealing actin filaments that moved along the cytoplasm. The expression of tight junction proteins at cell interfaces on the 14th day of seeding demonstrated the barrier function of epithelial cells on polymeric surfaces and the interaction between cells. Moreover, the expression of proteins crucial for retinal functions and matrix production was positively affected by fibrin, with an increment of PEDF. Our in vivo investigation with fibrin hydrogels revealed high short-term subretinal biocompatibility. Conclusions: The research of stem cell-based cell therapy for retinal degenerative diseases is more complicated than that of cell lines. Our results showed that fibrin is a suitable scaffold for hESC-RPE transplantation, which could be a new grafting material for tissue engineering RPE cells.

## 1. Introduction

The retinal pigment epithelium (RPE) is a layer of hexagonal cells located between the choriocapillaris and the retina’s photoreceptors. The RPE cells are critical for the growth, maintenance, and survival of adjacent photoreceptors and visual function [1]. Damage to the RPE may lead to photoreceptor degeneration and a range of retinal diseases, such as age-related macular degeneration (AMD) and retinitis pigmentosa (RP) [2]. People over 50 have an increased risk of retinal degenerative diseases. Visual loss in dry AMD is related to RPE metabolic disorder and photoreceptor geographic atrophy. So far, there is no effective treatment for dry AMD. Choroidal neovascularization (CNV) in wet-AMD can penetrate the Bruch’s membrane (BM) and damage the retinal structure. It is often treated with angiogenesis inhibitors such as anti-VEGF (anti-vascular endothelial growth factor). However, regular intravitreal injections of anti-VEGF could increase the risk of infection, retinal detachment, and even lens damage. Long-term administration and susceptibility to drug resistance or recurrence also burden patients’ with medical expenses. Therefore, there is an urgent need to explore novel therapies for retinal degenerative diseases. Various new technology evaluations have been carried out, including cell transplantation [3,4,5].

The RPE cells provides nutrition for photoreceptors, forms the blood-retinal barrier (BRB), and clears metabolites from the subretinal space by phagocytosing the outer segments of photoreceptors, thereby maintaining visual function. In contrast, retinal degenerative diseases are frequently associated with RPE dysfunction. Therefore, RPE cell transplantation may not only ensure its normal function by replacing the damaged cells but could also regulate the subretinal microenvironment, alleviate the subsequent photoreceptor degeneration and visual loss, and maintain retinal homeostasis.

Over the past few decades, numerous studies of RPE cell suspension injection into the subretinal space have demonstrated the promise of cell therapy. However, while the short-term results are the most effective, the long-term effects are often poor. This is probably related to the potential unresolved problems: 1. The suspension injection is prone to in situ leakages to form a proliferative membrane; 2. The percentage of attachment and survival of the suspended RPE injection is low; the maldistribution forms non-functional cell clusters, and the abundant apoptotic cells induce the microglia migration, local inflammatory response stimulation, and finally, reducing the graft efficacy; 3. Under aging or pathological conditions, RPE cells without adequate attachments may dedifferentiate into macrophages or fibroblasts through SMAD3, losing mature RPE function.

Effective therapy must simultaneously improve the defective RPE cells and the extracellular microenvironment. Therefore, the selection of suitable biomaterials and construction of the corresponding carrier scaffolds are crucial for retinal tissue regeneration. With the interdisciplinary application of clinical medicine, bioengineering, and the increasingly close medical–industrial collaboration, many studies have considered providing a transplanted cell-adhesive matrix so that the cells can maintain a stable microenvironment after transplantation. With the improvement of RPE transplantation by tissue engineering, cells are implanted and expanded in vitro on the surface of a single-layer degradable biomaterial scaffold, and then the whole graft is implanted into the subretinal space. The advantages of such RPE transplantation include organized polarized cell delivery and intact grafts with lower immunogenicity than dispersed cells.

Research and optimization of supporting RPE graft materials hold great promise. During development and subsequent implantation, synthetic polymer matrices serve as fundamental carriers for RPE cells. Currently, two polymers are under clinical testing, including polypropylene and polyester. These materials can be modified to form micropores and increase cell adhesion. However, laminin or vitronectin coating might be required to promote cell adhesion. Months after implantation, the polymer slowly degrades and becomes lodged between the RPE and the choroid, causing fibrin deposition and local inflammation in animal studies. Additionally, previous in vivo studies suggest that the rigidity of the material could damage the choroid.

Furthermore, the decreased choroidal permeability and low survival rate of the RPE cells are still a concern. However, hydrogel system materials display good biocompatibility, increased flexibility to fit tissue, a fast degradation rate, and excellent mechanical properties. With the close integration of tissue engineering, material science, and clinical medicine, we aim to screen common hydrogels for human embryonic stem cell-derived RPE cell transplantation and to reverse and regenerate retinas for future clinical applications.

As a naturally derived biomaterial, gelatin methacrylate (GelMA) and hyaluronic acid methacryloyl (HAMA) hydrogels are commonly used in medical regenerative engineering and tissue engineering [6]. The cellular effects of gelatin include propagating, diffusing, migrating, adhering, spreading, and activating cells [7]. Cartilage and vitreous tissue contain large quantities of hyaluronic acid (HA), which is pervasive in the extracellular matrix. Furthermore, HA exhibits good superelasticity, strength, and bio-compatibility in biomaterials. Alginate, an anionic polysaccharide derived from brown algae, is widely used in tissue engineering and cell encapsulation. Purified alginic acid membranes [8,9] and hydrogels [10,11,12] have been shown to enhance RPE cell growth and maintain specific functions. Moreover, the cross-linked fibrin network could form due to natural fibrinogen activation. Fibrin hydrogel is currently used clinically as a sutureless closure option during surgical incisions and is commercially produced using cGMP [13]. 

In vitro, several types of stem cells can differentiate into RPE cells, including adult stem cells, embryonic stem cells (ESC), and induced pluripotent stem cells (iPSC) [14]. Recently, human embryonic stem cell-derived RPE cells have been used clinically for the treatment of AMD and Stargardt disease [15,16]. Even though the long-term efficacy has not been determined, their safety in AMD treatment has been established. Previous RPE scaffold transplantations have mainly focused on cell lines, considering the unique characteristics of stem cell transplantation. Here, we evaluated four biodegradable hydrogels: GelMA, HAMA, alginate, and fibrin for ARPE-19 and hESC-RPE inoculations, respectively, as supports for adhesion, proliferation, and uniform distribution of differentiated RPEs in vitro. In addition, the subretinal biocompatibility of the hydrogels was evaluated in vivo. 

## 2. Materials and Methods

### 2.1. Culture of ARPE-19

ARPE-19 cells are an established but non-immortalized human RPE cell line obtained from the American Type Culture Collection (ATCC) (Manassas, VA, USA). The cells were grown in Dulbecco’s modified Eagle’s medium (DMEM)/F2 medium (Gibco, Carlsbad, CA, USA) containing 3 mM L-glutamine (Gibco, Carlsbad, CA, USA), 10% fetal bovine serum (Gibco, Carlsbad, CA, USA), 100 U/mL penicillin, 100 μg/mL streptomycin, and incubated at 37 °C in a humidified environment of 5% CO_2_ and 95% air.

### 2.2. Culture of hESC-RPEs

To stimulate hESC-RPEs development, xeno-free Essential 8 culture Q-CTS-hESC-2 Cell Line media (Gibco, Carlsbad, CA, USA) were employed, as previously described [16]. In brief, the differentiation of hESCs to RPEs used steps of hyperfusion, acquired pigment foci, and excision. The medium for RPEs spreading from excised pigment foci contained 77% KO-DMEM-CT (Invitrogen, Carlsbad, CA, USA), 20% knockdown serum-free xenograft CT (Invitrogen, Carlsbad, CA, USA), 1% CTS-glutamine-1 supplement (Invitrogen, Carlsbad, CA, USA), 1% MEM-NEAA (Invitrogen, Carlsbad, CA, USA) and 1% 2-mercaptoethanol (Procell, Wuhan, China). Human embryonic stem cell-derived RPE cells were cultured in cell culture dishes at 37 ℃ in an incubator with 5% CO_2_/95% air with the medium changed every 2 days. Proliferating cultures were digested by TrypLE Express (Gibco, Carlsbad, CA, USA) and then passaged 1:4. Additionally, hESC-RPE was evaluated according to morphological characteristics, presence of pigments in cells, bestrophin-1, CRALBP, MITF, and PAX6 immunostainings (BD Biosciences, San Jose, CA, USA).

### 2.3. Synthesis of GelMA

GelMA was prepared as previously reported [17]. Briefly, a beaker with a magnetic stirrer was filled with 100 mL of PBS, and 20 g of gelatin (Sigma, St. Louis, MO, USA) was added and completely dissolved in the solution at 60 °C. Methacrylic anhydride (2 mL, Sigma, St. Louis, MO, USA) was added slowly and stirred vigorously, and the emulsion was rotated at 60 °C for 3 h. Unreacted methacrylic anhydride was removed using dialysis membranes (12–14 kDa, Solarbio, Beijing, China). Subsequently, the reaction products were freeze-dried.

### 2.4. Synthesis of HAMA

To prepare HAMA, 1 g of sodium hyaluronate (Bloomage Biotechnology, Hong Kong, China) was dissolved in 100 mL distilled water, then 1 mL methacrylic anhydride was added to reach a final concentration of 1% (*v*/*v*) and reacted for 24 h (4 °C). Then, 5M sodium hydroxide was added to maintain the reaction solution (pH 8–10). After the reaction, the solution was dialyzed at 4 °C for 3 days. The dialysis bag solution was frozen at −80 °C for 3 h and then freeze-dried for 2 days to obtain HAMA.

### 2.5. Synthesis of Modified Alginate

Sodium alginate (Sigma, St. Louis, MO, USA) was transformed with the G_4_RGDY peptide sequence (Holder, Wuhan, China) containing the RGD amino acid sequence using carbodiimide chemistry [18]. To activate the sodium alginate polymer chain’s carboxylic acid, 1-ethyl-(dimethylaminopropyl) carbodiimide (EDC; Aladdin, Shanghai, China) was used. Then N-hydroxy sulfosuccinimide (Aladdin, Shanghai, China) and peptides were added. Hydroxylamine hydrochloride was added 20 h later to quench the process. Subsequently, the sodium alginate solution was dialyzed in decreasing salt solution for 3 days (MWCO 3500, Solarbio, Beijing, China) and then freeze-dried.

### 2.6. Compressive Measurements

The compressive modulus of several hydrogels was determined using stress measurements. Following ISO 7743/ISO 527-2, cylinder-shaped plastic molds were created, and UV-cured hydrogel specimens were inserted into the molds. Hydrogels were examined with the MTS Exceed model E43 mechanical tester at a 1 mm/min rate. The compressive modulus was calculated based on the slope of the linear region (0–5% strain).

### 2.7. Formation of Different Thin-Layer Hydrogels

There were seven groups of different concentrations of hydrogels as cell growth substrates: 2% (*w*/*v*) GelMA, 5% (*w*/*v*) GelMA, 1% (*w*/*v*) HAMA, 2% (*w*/*v*) HAMA, 1% (*w*/*v*) sodium alginate, 2% (*w*/*v*) GelMA + 1% (*w*/*v*) HAMA and 0.5% (*w*/*v*) fibrin. GelMA and HAMA films were prepared with DPBS and 0.2% (*w*/*v*) lithium phenyl-2,4,6-trimethylbenzoylphosphonate (LAP, Sigma, St. Louis, MO, USA) and then cross-linked with UV for 15 s (360 nm, Run LED, Shanghai, China). Sodium alginate hydrogels were prepared with 1% *w*/*v* RGD sodium alginate, 50 mM Ca-EDTA and 0.1% (*v*/*v*) acetic acid, and the fibrinogen gel was made by mixing fibrinogen (Haikon, Shanghai, USA) and thrombin (Sigma, St. Louis, MO, USA, 10 U/mL).

### 2.8. Cell Morphology, Proliferation, and Viability in Different Hydrogels

ARPE-19 cells were plated on each hydrogel membrane and polyester tissue culture polystyrene (TCP; Costar, Tehama, CA, USA) as control at a density of 1 × 10^5^ cells/cm^2^. Meanwhile, hESC-RPE cells were inoculated onto each membrane with a density of 1 × 10^5^ cells/cm^2,^ and ovalbumin (VTN, Gibco, Carlsbad, CA, USA) was used as a control. The culture medium was changed every two days. After seven days, the vitality of ARPE-19 and hESC-RPE cells on the hydrogels was assessed by live/dead assay using the LIVE/DEADs kit (Invitrogen, Carlsbad, CA, USA) to determine the influence of six groups of hydrogels with varying concentrations prepared as substrates for RPE growth. Images were captured with an inverted fluorescence microscope (Olympus, Tokyo, Japan). Furthermore, to assess the cell growth of RPE on different hydrogels, the DNA content of RPE was measured at 1, 3, 5, 7, 9, 12, and 14d using the double-stranded DNA HS analysis kit from Qubit (Yeasen, Shanghai, China).

### 2.9. hESC-RPEs Characterization by Immunostaining

hESC-RPEs were inoculated at a density of 1 × 10^5^ cells/cm^2^ onto selected hydrogels and VTN for 14 ± 3 days. Cells were washed twice with PBS and fixed with 4% paraformaldehyde (PFA) for 20 min. Later, hESC-RPEs were treated with RPE-65 (MA1-16578, Invitrogen, Carlsbad, CA, USA) and ZO-1 (33-9100, Invitrogen, Carlsbad, CA, USA) overnight at 4 °C, followed by incubation with Alexa Fluor 488-labeled goat anti-rabbit (A-11008, Invitrogen, Carlsbad, CA, USA) in the dark at room temperature for 1 h. DAPI was used 10 min after the 1 h incubation. A confocal laser scanning microscope was used to study the stained cells (FV3000, Olympus, Tokyo, Japan).

### 2.10. Secretion Ability of PEDF and VEGF by ELISA

hESC-RPEs were inoculated at a density of 1 × 10^5^ cells/cm^2^ on the selected hydrogel and VTN as controls for 14 ± 3 days. Pigment epithelium-derived factor (PEDF) levels in supernatants were measured using the PEDF ELISA kit (BioVendor Research, Czech Republic). Vascular endothelial growth factor (VEGF) levels were measured with a VEGF ELISA kit according to the manufacturer’s instructions (Abcam, Cambridge, UK, USA) [19].

### 2.11. Subretinal Transplantation of Selected Hydrogel

Animal experiments were conducted according to the guidelines of the Statement for the Use of Animals in Ophthalmic and Vision Research (ARVO) and approved by the Animal Care and Use Committee of Dalian Medical University (No. 20190302-42). This research employed 20 mature C57BL/6J mice (males and females, 25 ± 5 g). All mice were obtained from the Dalian Medical University Animal Care Center, individually kept in a 12 h light/dark cycle, and fed with regular mouse food and water at room temperature (25 ± 5 °C). The subretinal space of the right and left eyes were transplanted with the selected hydrogel or control PBS, as previously described [20]. Mice were rendered unconscious by intraperitoneal injection of 3 mL/kg of 10% chloral hydrate. The pupils of both eyes were dilated using tropicamide eye drops. After incising the dorsolateral portion of the bulbar conjunctiva, the 29G needle was inserted 3 mm posterior to the corneal rim to pierce the sclera. The hydrogel was maintained chilled during the operation. Only 1 μL hydrogel was progressively injected into the subretinal space using a microsyringe. The needle was maintained for 30 s before being removed extremely carefully to minimize hydrogel leakage. The retina was examined through the glass coverslip contact lens. The spherical protrusion on the retina indicated a successful injection. Postoperatively, all animals were given 210 mg/L cyclosporine-containing water, and tobramycin/dexamethasone ointment eye drops.

### 2.12. In Vivo Degradation of the Selected Hydrogel

The degradation of the selected hydrogels was followed in vivo by measuring the thickness of the hydrogels over time. Lethal intraperitoneal injections of sodium pentobarbital were administered to animals at 1, 7, and 14 days. Eyes were removed and placed in 4% PFA. The anterior segment was excised, and eyecups were post-fixed in 4% PFA for 2 h and 30% sucrose/PBS overnight. The tissue was dehydrated, cleared, and embedded in paraffin. Paraffin-embedded tissue blocks yielded 10 mm tissue slices. Sections were rinsed in xylene to remove paraffin, rehydrated in ethanol, and then washed in distilled water. Hematoxylin and eosin (HE)-stained sections were imaged under the microscope (IX71, Olympus, Tokyo, Japan).

### 2.13. Statistical Analysis

All statistical analyses were conducted by SPSS 13.0 (SPSS, Inc., Chicago, IL, USA). All data were represented as mean ± standard deviation. A one-way analysis of variance (ANOVA) was utilized to compare the differences between different groups. A *p*-value below 0.05 was considered statistically significant.

## 3. Results

### 3.1. Identification of hESC-Derived RPE Cells

**Figure 1:**
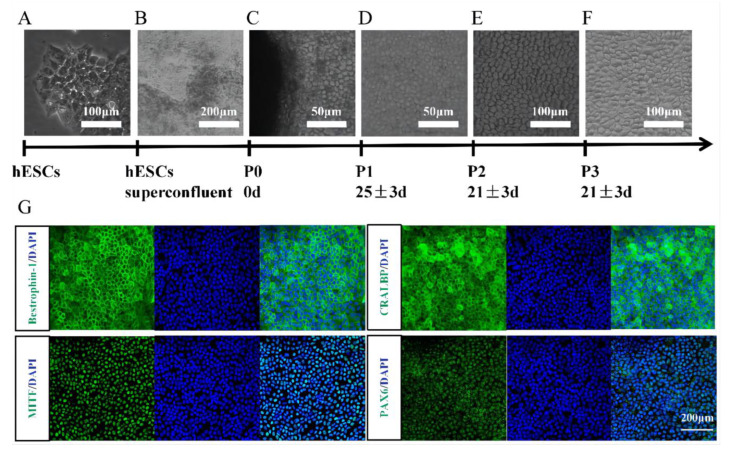
High purity and good differentiation of hESC-RPE. (**A**) hESCs (Q-CTS-hESC-2). (**B**) ESCs were perfused after pigment granule deposition. (**C**) Isolation of pigment foci as the beginning of differentiation of hESC-RPE cells. (**D**) RPEs showed the typical cobblestone-like morphology, as well as pigment accumulation. (**E**) Second generation hESC-RPE. (**F**) Third generation hESC-RPE. (**G**) Immunostaining of bestrophin-1, CRALBP, MITF, PAX6 in hESC-RPE cells (third generation).

We used the human embryonic stem cell line (Q-CTS-hESC-2) as a cell source to induce RPE cell differentiation [21] (Figure 1A). The hESC colonies became superfused after 7 days of spontaneous differentiation and formed pigmented foci after approximately 25 days (Figure 1B). After 35 ± 5 days, the pigmented foci were sufficient to be removed and seeded into 6-well plates, enabling RPE cells to adhere and proliferate. hESC-RPE differentiation began on the day the pigmented foci were isolated [16] (Figure 1C). After day 20, RPE cells exhibited pigment accumulation and a typical cobblestone morphology (Figure 1D). Immunostaining for bestrophin-1, CRALBP, MITF, and PAX6 in hESC-RPE cells demonstrated high purity and good differentiation (Figure 1G).

### 3.2. Evaluate the Mechanical Properties of the Low Concentration Hydrogels

**Figure 2:**
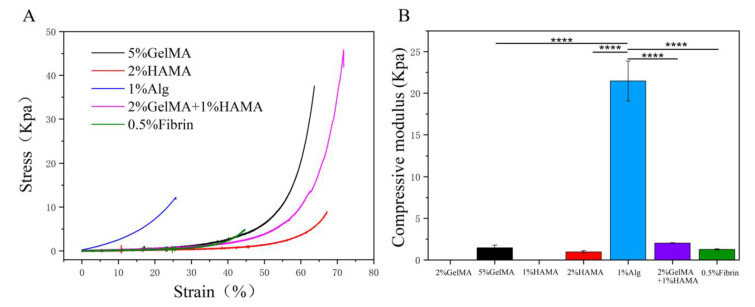
Evaluating the mechanical properties of the low concentration hydrogels. (**A**) Stress–strain curves for hydrogels under compression. (**B**) Corresponding compressive moduli of hydrogels. ****: *p* < 0.0001.

The percentage of hydrogel needs to guarantee gelation as low as possible. Rheology and compression tests (modulus E) were used to evaluate the mechanical properties of seven groups of hydrogels. Among them, the strength of 2% GelMA and 1% HAMA was too low to be bonded in the machine. The compression tests showed similar stress–strain curves for the different hydrogels, with an initial linear increase in stress with increasing deformation at small strains and an exponential increase in stress at constant strain at large deformations (Figure 2A). The hydrogels of the five groups presented significantly different stiffnesses, with compressive moduli of 1.46 ± 0.31 kPa for 5% (*w*/*v*) GelMA gels, 0.96 ± 0.13 kPa for 2% (*w*/*v*) HAMA gels, 21.46 ± 2.4 kPa for 1% (*w*/*v*) Alg-RGD gels, 2.02 ± 0.04 kPa for 2% (*w*/*v*) GelMA and 1% (*w*/*v*) HAMA gels, 1.2 ± 0.06 kPa for 0.5% (*w*/*v*) fibrin gels (Figure 2B). Since 1% (*w*/*v*) Alg-RGD hydrogels had a significantly higher percentage yield strain than the other hydrogels, these findings allowed us to prepare soft hydrogels by determining specific low concentrations of 5% (*w*/*v*) GelMA, 2% (*w*/*v*) HAMA, and 0.5% (*w*/*v*) fibrin, respectively.

### 3.3. The Normal Cell Morphology, Proliferation, and Viability of hESC-RPE on Fibrin

**Figure 3:**
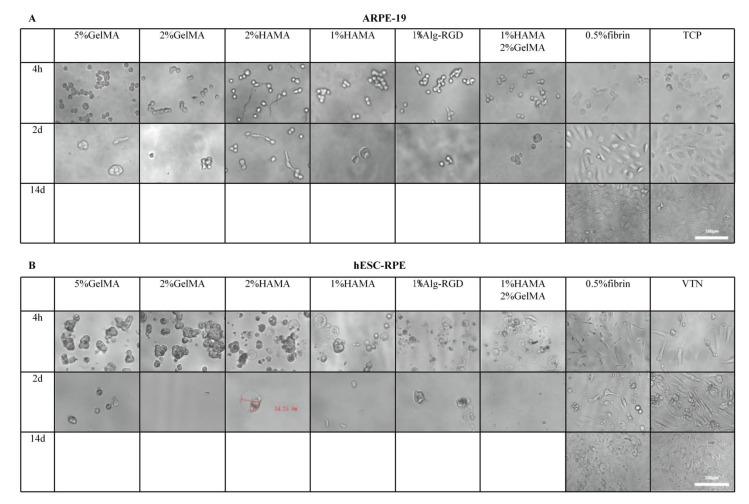

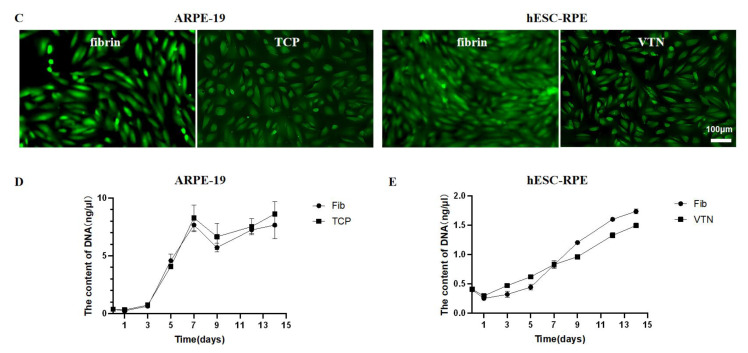
The seven groups of hydrogels with different concentrations prepared as the substrates for ARPE-19 as well as hESC-RPE cell growth. Only fibrin protein hydrogels allowed cells to form a uniform distribution at low concentrations, while other types of hydrogels formed cell aggregates without adhesion and were easily washed off after two days. (**A**) Optical images of ARPE-19 on different hydrogels. (**B**) Optical images of hESC-RPEs on different hydrogels, while damaged cells with special vesicles were measured by the red bar. (**C**) Representative confocal microscopy images of RPE on fibronectin, which was stained with calcein and ethylene dimer for live/dead assay. (**D**) The DNA content of ARPE-19 on fibrin as well as the TCP control. (**E**) The DNA content of hESC-RPEs on fibrin as well as the VTN control.

We next examined the cell morphology, proliferation, and viability of ARPE-19 and hESC-RPE cells on different hydrogels in vitro. After the cells were attached to the surface, their distribution was observed using light microscopy. Cells spreading on fibrin showed a uniform distribution similar to the controls. In contrast, other hydrogels formed cell aggregates without adhesion, which were easily washed out after two days (Figure 3A,B). Only fibrin (0.5%) resulted in a similar cell adhesion, morphology, and proliferation of hESC-RPE and ARPE-19 cells compared to the commercial VTN and TCP controls. In contrast, hESC-RPEs on other hydrogels formed additional vesicles, which were thought to be characteristics of apoptosis (Figure 3B, red bars). The ARPE-19 and hESC-RPE cells cultured on fibrin maintained tight junctions, which were pigmented and formed a cobblestone monolayer of cells. (Figure 3A,B; 14 days cell culture). As seen above, diluting the hydrogels caused the viscosity to drop dramatically (Figure 2) and could explain the inability of the majority of low-concentration solutions to facilitate uniform cell distribution and adhesion. Live/dead cell labeling also demonstrated that ARPE-19 and hESC-RPE cells survived on fibrin hydrogel (Figure 3C). Moreover, the DNA content measurements showed a dramatic increase in ARPE-19 proliferation on fibrin, especially during the first 5 days and then stabilized after day 7. The DNA content on day 5 was eight times higher than on day 1 (Figure 3D). Meanwhile, the DNA content of hESC-RPE cells continued to increase slowly for nearly 2 weeks; the DNA content on day 14 was 6 times higher than that of day 1 (Figure 3E). These results suggest that fibrin hydrogels provide a physiologically relevant microenvironment to support hESC-RPE survival and proliferation.

### 3.4. Functional Protein Secretion of hESC-RPE on Fibrin

**Figure 4:**
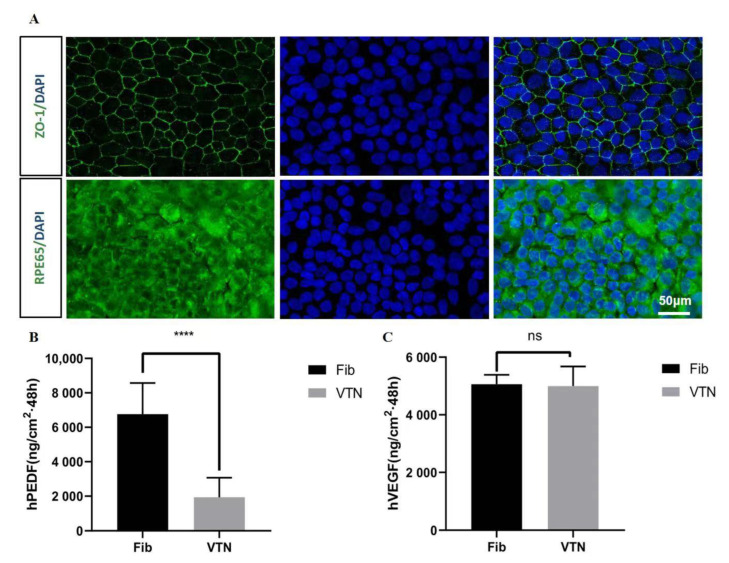
hESC-RPE matured over 2 weeks, and functional proteins secreted on fibrin. (**A**) hESC-RPE cells were cultured on fibrin. Representative phase and immunostained images are shown for ZO-1, RPE-65 expression. (**B**) ELISA of hESC-RPE against PEDF after differentiation (*n* = 3). (**C**) ELISA of hESC-RPE against VEGF after differentiation (*n* = 3): ****: *p* < 0.0001.

The biological functions of hESC-RPE on fibrin hydrogels are essential for practical applications and studying cell behavior. ZO-1 is a tight junction protein that plays a critical role in the formation of epithelial cell polarity and is an important marker of epithelial cell differentiation. At the same time, RPE-65 is positively related to the maturity of RPE cells. Immunofluorescence staining showed a positive expression of ZO-1 in RPE cells cultured with fibrin (the intercellular junction was green), with a hexagonal pattern in most cells. RPE-65 expression was also positive (Figure 4A). We then evaluated the secretion capability of PEDF using ELISA. The level of PEDF secretion in hESC-RPE cells on fibrin (6764 ± 2448 ng/cm^2^, at 48 h) was significantly higher than on VTN (1936 ± 1861 ng/cm^2^, at 48 h). hESC-RPE cells on fibrin and VTN did not differ in VEGF secretion (Figure 4C). These data indicate that hESC-RPEs on fibrin partially increase the secretory function of hESC-RPE cells to produce the PEDF growth factor, enhancing RPE survival and facilitating retinal cell differentiation and maturation.

### 3.5. In Vivo Immunogenicity and Degradation of Fibrin in the Subretinal Space

**Figure 5:**
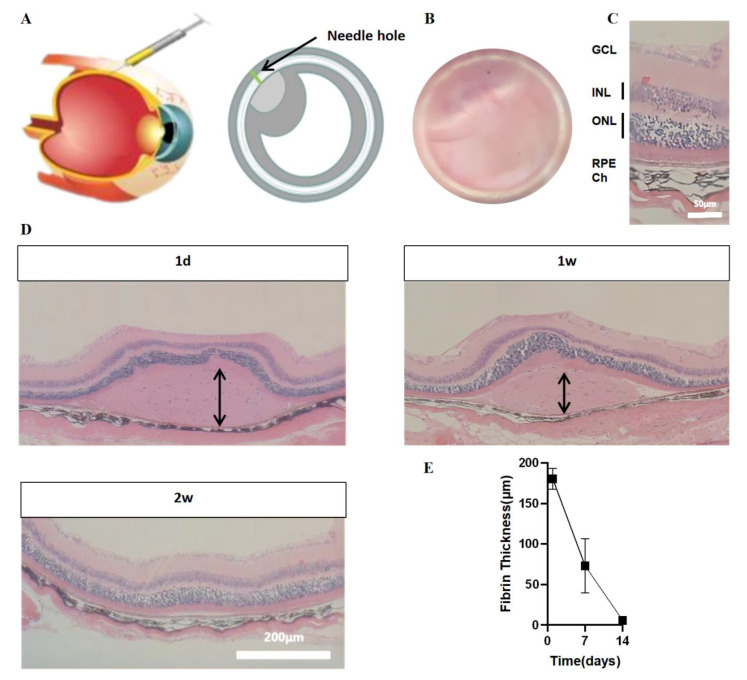
Immunogenicity and degradation of fibrin in the subretinal space. (**A**) Schematic of the subretinal space injection. (**B**) Macroscopic view of injected fundus of a mouse eye. (**C**) Close up image of HE stained histological sections where the injection was administered, revealing the healthy retina. (**D**) Micrographs of HE-stained tissue sections of animals at 1, 7 and 14 days. The fibrin implants were eosinophilic and most of the gel was retained for 1 week. After two weeks, fibrin gels were no longer evident. Both time periods reveal a healthy neuroretina inside the implanted location (arrows indicate fibrin). (**E**) Graph showing the change in fibrin protein thickness over time in animals. Thickness indicates remaining fibronectin implants. GCL: ganglion cell layer. ONL: outer nuclear layer. INL: inner nuclear layer. RPE: retinal pigment epithelium. Ch: choroid.

A schematic diagram of the subretinal injection via the external route is shown in Figure 5A. We used a microsyringe at 2 mm behind the corneal limbus to pierce the bulbar wall slightly parallel to the sclera obliquely and injected about 1 μL solution into the right eye. If the local retina of the fundus showed a circular bulge under the operating microscope, the injection was considered successful (Figure 5B). HE staining confirmed the existence of hydrogel 1 day after implantation. Subsequently, the HE slices were examined to verify fibrin gel degradation and retinal integrity in the implant area (Figure 5D). Retinotomy and the head of the optic nerve were used to locate the implant. At the location of the retinotomy, a scar thicker than the surrounding retina was discovered. One week after implantation, eosinophilic hydrogel appeared distal to the retinotomy site. The neurosensory retina on the gel was found to be in good condition with a proper anatomical structure and outer photoreceptor segments in contact with the fibrin hydrogel. Furthermore, we did not find any roseola node formation or immune cell infiltration. The fibrin appeared to retain a relatively smooth outside surface. The fibrin was degraded within 14 days (Figure 5E). These were mainly confined to the weakening of the gel’s outer edge after one week.

## 4. Discussion

This work aimed to identify a scaffolding or covering material suitable for the implantation of hESC-RPE cells during subretinal transplantation. We screened hydrogels that would aid in adhesion and differentiation, offer sustained survival for surgical implantation of hESC-RPE cells, and disintegrate rapidly within several days. Our results showed that fibrin is suitable for this application.

Damage to the BM and RPE cell layers, such as stromal damage and drusen deposition, are important causes of retinal diseases, including early AMD [22]. It is difficult for donor RPE cells to attach to the recipient’s diseased BM during RPE transplantation. Even if they can be reattached to the recipient’s lesioned BM, the differentiation function of the transplanted cells is significantly limited [23]. Therefore, the quality and quantity of BM that provides a scaffold for cell attachment could determine the fate of RPE grafts [24].

Injecting retinal progenitor cell (RPC) suspension leads to the survival of only a few cells [25]. Biodegradable scaffolds enhance transplanted cell survival. The optimal BM replacement is one of the keys to the success of RPE transplantation; it should be biocompatible, biodegradable, and bioabsorbable and guide proper cell adherence, differentiation, and proliferation [26]. Traditionally, solid scaffolds comprised of synthetic polymers such as poly(L-lactic acid)/poly(lactic-co-glycolic acid) (PLLA/PLGA), poly(3-caprolactone) (PCL), and poly(glycerol-sebacate) (PGS) have been used for this purpose [27]. These scaffolds are rigid, and their implantation in the subretinal space is invasive and may result in retinal detachment [28,29]. Recent research has focused on biodegradable hydrogels as deliverable scaffold systems for stem cells [30]. Hydrogel has the advantage of high water content, which can wrap cells with a similar structure to the extracellular matrix, permeabilize nutrients, and is less invasive than solid scaffolds [31,32,33].

This study investigated four biodegradable hydrogels as cell growth carriers: GelMA, HAMA, sodium alginate, and fibrin. ARPE-19 and hESC-RPE were inoculated and evenly distributed on these hydrogels and constructed to mimic retinal adhesion, proliferation, and growth of BM functionality. Although all materials were derived from natural sources, our hydrogel scaffolds were relatively thick and did not contain the five layers of natural BM. Therefore, our chosen hydrogel might sustain RPE cells like natural BM but without the biophysical cues. GelMA is commonly used for cell culture and tissue engineering scaffolds [34,35]. Photocrosslinking improves GelMA stability at physiological temperatures and permits fine-tuning of mechanical characteristics [36,37]. Hyaluronic acid has limited cell adhesion and has been modified with collagen, fibrin, RGD peptide, and gelatin. One study combined GelMA and HAMA to seed human umbilical vein endothelial cells (HUVECs). This hydrogel combination proved promising for cardiovascular tissue engineering [38].

RGD-alginate hydrogels have been used to transplant rat fetal retinal tissue [39] and stimulate neural differentiation in mouse ESCs [40]. Alginate preserves primary and adult hRPE cell viability [10,12]. Encapsulation in 1% alginate hydrogel enhances the pigmented RPE phenotype of human, porcine primary adult RPE cells and the expression of RPE markers such as RPE65 and tyrosinase. Meanwhile, the 3D culture of hPSCs in RGD-alginate hydrogel enhances the formation of retinal tissue [11]. In addition, sodium alginate and hyaluronic acid are used in ophthalmic products, including intraocular products [41], and are well tolerated by the eye. However, protein-based materials should have low concentrations to achieve a controlled onset of degradation and degradation rates within days to months after subretinal injection. There is evidence of ultrastructural and cellular damage in the inner retinal layers as well as pre-retinal hemorrhage with collagen-based support materials [42,43]. Fibrinolytic enzymes are routinely clinically used in the eye, unlike other hydrogels. Ocriplasmin and alteplase come in various doses and have mild retinal side effects [13,44]. Moreover, fibrin-protein hydrogels containing peptidases are safe and degradable scaffolds for the subretinal implantation of iPSC-RPE [45].

There have been several successful clinical trials of hESC-RPE cell suspension transplantation, which could potentially treat AMD. Although several promising hydrogels have been successfully used for the multiple biomedical applications described above, few studies have investigated cellular behavior and the tissue responses to hydrogels as RPE growth vehicles for hESCs. However, the introduction of RPE cells in implants generates xenografts and thus triggers an immune response. Therefore, low concentrations of degradable scaffolds are urgently needed for rapid hydrogel degradation and successful integration. In our study, we compared the growth of hESC-RPE and ARPE-19 cells on seven groups of four biodegradable hydrogels with different concentrations. We found no significant difference in the adhesion rates of the RPE cells between the fibrin and conventional VTN groups after 4 h of inoculation at the same density. In contrast, the adhesion rate of the other materials was significantly lower. Moreover, changing the medium two days after inoculation, the hESC-RPE cells on most scaffolds, other than fibrin, were washed away due to their non-adherence with the scaffold. The contact of HAMA with hESC-RPE also showed a change in cell morphology, which may be related to apoptosis. It indicated that fibrin was more suitable for the adherent growth of hESC-RPE and ARPE-19 cells. In the cell proliferation experiment, we found that the number of adherent cells in the fibrin group was fewer than that in the control VTN group on the first day after inoculation; however, the proliferation in the fibrin group was significantly faster than that in the control group on the seventh day. On day 14, cell proliferation peaked in both groups, with no significant difference between the fibrin and control groups. Notably, the number of hESC-RPE cells in the fibrin group was still lower than that in the control group on day 1, which may be due to the ability of cells to adhere and proliferate. Some academics argue that the proliferation ability of hESC-RPE cells on different supports is related to the structure of the matrix itself and the interaction between cells and the matrix, including the morphology and chemical structure of the matrix membrane. The mechanical differences between the hydrogels could explain the difference in the adhesion and proliferation of RPE cells; the mechanical properties of fibrin were similar to BM. However, increased cell adherence does not guarantee cell proliferation and differentiation ability. Therefore, further immunofluorescence and ELISA experiments should be conducted for relevant verification.

ZO-1 is a tight junction protein that plays an essential role in the formation of epithelial cell polarity and is an important marker of epithelial cell differentiation, while RPE-65 is positively related to the maturity of RPE cells. As a type of epithelial cell, differentiated RPE cells usually have polarity, and tight junction functions constitute the outer barrier of the retina. The expression of ZO-1 in RPE cells and the arrangement of the RPE monolayer in vitro were consistent with the characteristics of the normal differentiation of RPE cells in vivo. We found that on the 14th day of culture, hESC-RPE cells on both fibrin and control groups formed RPE monolayers, ZO-1 staining was complete and orderly, and most of them surrounded a single hESC-RPE cell. These observations suggested that hESC-RPE cells on fibrin maintain typical epithelial differentiation characteristics and have polarity and tight junction function. Moreover, overexpression of RPE-65 was strongly related to the proper differentiation and high purity of hESC-RPE cells on fibrin. Additionally, a variety of cytokines, such as PEDF and VEGF, are secreted by the RPE cells. hESC-RPE cells cultured on fibrin secreted more PEDF than control VTN, enhancing RPE survival and facilitating retinal cell differentiation and maturation [46]. Studies show that PEDF plays a neuroprotective role on retinal cells in the pathogenesis of neurodegenerative diseases. We selected healthy adult C57BL/6J mice and implanted fibrin and PBS in the subretinal space without cells. Histopathological sections found that although there was a certain degree of foreign-body reaction (monocyte infiltration), fibrin was generally accepted by the subretinal space of healthy adult C57BL/6J mice without apparent rejection. Therefore, preliminary studies indicate that fibrin hydrogel has general biocompatibility in the subretinal space.

In this study, we selected typical biodegradable natural hydrogels with good biocompatibility for hESC-RPE implantation. Using scaffold-free RPE microtissue delivery [47] or even microcarriers as carriers for subretinal cell transplantation [48,49], the use of biogel materials should not be limited to monolayer cell transplantation scaffolds. We had previously conducted several studies related to microgel cell transplantation. and attempted to make it feasible, for the first time, to use microgels as injectable fillers for retinal cell transplantation for the treatment of retinal diseases. Therefore, the premise of our material optimization was to ensure that it was conducive to cell adhesion, differentiation, and growth as a classy coating material, regardless of conditions qualifying it as a monolayer graft scaffold of graft hardness, thickness, and so on. Considering that the introduction of implants into RPE cells will lead to xenotransplantation, which results in an immune response, low concentration is an urgent need for the rapid degradation and successful integration of hydrogels. We further used conventional compression tests to evaluate the mechanical properties of the hydrogels. After selecting fibrin as the growth scaffold for hESC-RPE transplantation, considering the different ways of future transplantation and the different purposes of usage, we did not combine fibrin with cells or make a monolayer cell scaffold complex for transplantation; we only carried out pure fibrin hydrogel transplantation to explore the degradation and evaluate its biocompatibility for further animal experiments. Nevertheless, to understand the use of hydrogels for intraocular transplantation to optimize the biological function of transplanted cells, in the anticipation of further design and embellishment of the material properties to combine cells to double-promote disease treatment, the simple fibrin hydrogel material is still limited. Therefore, it is a crucial issue that needs to be resolved using further animal experiments and clinical studies. Future studies should also investigate the scaffold as a dual-use platform, combining regenerative cells to deliver drugs and biologics to ocular tissue.

## 5. Conclusions

In this study, we investigated four typical biodegradable and biocompatible hydrogels as supports for hESC-RPE subretinal transplants as a potential treatment for AMD. hESC-RPE cells retained the cobblestone-like morphology, specific protein expressions, polarized morphology, and maturation-related functional PEDF/VEGF secretion capability only with the fibrin hydrogels. After degradation of the fibrin hydrogel within 2 weeks, the retina appears to reattach to the underlying RPE. To our knowledge, this is the first report on screening typical biodegradable hydrogels for RPE adhesion and proliferation of hESCs. Our data suggest that biodegradable fibrin hydrogels have suitable mechanical properties for easy transscleral-driven subretinal implantation and can be considered biocompatible scaffolds for functional hESC-RPE subretinal transplantation.
